# Association of SNPs rs1501299 and rs17300539 in *ADIPOQ* with Serum Adiponectin Level and risk of Polycystic Ovarian Syndrome (PCOS) in population of Southern Punjab, Pakistan

**DOI:** 10.12669/pjms.41.6.10872

**Published:** 2025-06

**Authors:** Amna Mushtaq, Asia Bibi, Sajid Malik, Nahid Kausar

**Affiliations:** 1 Amna Mushtaq, PhD, Department of Zoology, The Women University Multan, Multan, Pakistan; 2 Asia Bibi, PhD, Department of Zoology, The Women University Multan, Multan, Pakistan; 3 Sajid Malik, PhD Human Genetics Program, Department of Zoology, Quaid-i-Azam University, Islamabad, Pakistan; 4 Nahid Kausar, PhD, Department of Zoology, The Women University Multan, Multan, Pakistan

**Keywords:** Association study, Consanguinity, Metabolic disorder, Obesity, Polymorphisms, infertility

## Abstract

**Objectives:**

Polycystic Ovarian Syndrome (PCOS) is a common endocrine and metabolic disorder, and a major health concern in Pakistani women, causing infertility, obesity, and psychological complications. The current study was aimed to elucidate the risk factors for PCOS and the association of SNPs rs1501299 and rs17300539 in Adiponectin (*ADIPOQ*) gene with serum adiponectin level and the vulnerability of PCOS in women ascertained from Southern Punjab, Pakistan.

**Methods:**

In this observational case-control study design, 204 clinically confirmed PCOS patients and 150 healthy controls were recruited during 2020-2022 from tertiary care hospitals in Southern Punjab, Pakistan. SNPs were genotyped by ARMS-PCR and RFLP methods.

**Results:**

The analyses revealed that obesity, smoking, family history, parental consanguinity, environmental exposures and ethnicity were significant risk factors of PCOS. SNPs (rs1501299 and rs17300539) were found to be significantly associated with elevated risk of PCOS in different inheritance models. At rs1501299, G-allele confers a significantly high risk of PCOS. Further, G-G haplotype at the forward strand and G-A haplotype at the reverse strand were significantly highly prevalent among the individuals with PCOS. Serum adiponectin level was significantly lower in individuals with GG genotype at rs1501299.

**Conclusion:**

This study elucidated certain bio-epidemiological variables as risk factors of PCOS. Additionally, SNPs *rs1501299* and *rs17300539* were strongly associated with increased PCOS risk. These findings may have direct application to clinical practice in women health. Additional studies are warranted to identify panel of SNPs that may be utilized as potential biomarkers for the risk of PCOS in this population.

## INTRODUCTION

Polycystic Ovarian Syndrome (PCOS), a common endocrine, metabolic disorder that affects women of fertile age, is characterized by hormone imbalance and irregular menstrual periods.^1^,^2^ The additional symptoms are weight gain, excess hair growth and insulin resistance which lead to metabolic disorders and cardiovascular problems.^2^,^3^ PCOS is a leading cause of infertility in women. It may start during adolescence, but symptoms may fluctuate over time. PCOS women are prone to many additional socio-psychological problems, marital maladjustment and morbidity.^4^

Etiologically, PCOS is a very heterogeneous condition with strong environmental and epigenetic influences including dietary and other lifestyle issues.^3^ PCOS may also manifest in genetically susceptible females following exposures to environmental and nutritional factors.^2^ In certain instances, PCOS may run in families. However, in most cases, PCOS has a polygenic and multifactorial syndromic presentation. Data accumulated over the years have demonstrated that many genes are associated with PCOS. However, independent studies carried out on PCOS women from multiple families have not been successful in finding fully penetrant variant(s).^5^

Adiponectin (*ADIPOQ* or ACRP30) is the most abundant protein that is secreted by adipose tissues. Adiponectin is involved in metabolic processes including adipose tissue catabolism and glucose level.^6^ Some research findings proposed that higher prevalence of SNP rs1501299 (276 G>T) variant of *ADIPOQ* is associated with the risk of pathogenesis of PCOS.^7^,^8^ Genetic association studies on PCOS in the Pakistani population are scarce.^9^,^10^ Particularly there are no data published on the genetic predisposition of PCOS in Southern Punjab.

Towards this end, the current study aimed to investigate the association of SNPs rs1501299 and rs17300539 with the pathogenesis of PCOS in a cohort assembled from Southern Punjab, Pakistan. The SNPs rs1501299 (in intron two) and rs17300539 (in the promoter region) of the *ADIPOQ* gene have been implicated in metabolic disorders such as obesity, Type-2 diabetes, and insulin resistance—conditions frequently comorbid with PCOS. However, the findings across different populations remain inconsistent, possibly due to ethnic variations in genetic architecture or other confounding factors. Hence, this research has a significant clinical relevance, as identifying genetic markers associated with PCOS could improve early diagnosis, risk stratification, and personalized treatment approaches for affected women in South Punjab, Pakistan, and similar populations.

## METHODS

### Subjects recruitment:

A total of 354 subjects (204 patients; 150 controls) were recruited. PCOS patients (n=204, here referred to as cases) were recruited from various tertiary care hospitals across Southern Punjab, Pakistan.^4^ This study was carried out during 2020-2022.

Seventy-three cases were recruited from Bakhtawar Amin Hospital Multan, 47 from Nishtar Hospital Multan, 31 from DHQ Muzaffargarh, 21 from Indus Hospital Muzaffargarh, 19 from THQ Jatoi, and 13 from private clinics. The consenting patients were randomly selected from those attending the gynecology/endocrinology clinics, and there was no preference on the grounds of ethnicity, language, or literacy. The diagnosis of PCOS individuals was based on the Rotterdam criteria.^11^ The subjects suffering from renal and liver dysfunction, thyroid disorders, hyperprolactinemia, and Cushing’s syndrome, were not included. Moreover, females with a history of hysterectomy, pregnant or lactating women, or with prior hormonal therapy were also excluded. The patients not having permanent residence in Southern Punjab were excluded.

The healthy control group (n=150), ascertained from general public, comprised women with regular menstruation and without any clinical manifestations. The individuals were informed about the study aim, and written consent was collected from each respondent, followed by the collection of demographic data. Data were collected on BMI, history of smoking (non-smoker, passive smoker), family history (negative, positive), parental consanguinity (unrelated marriage, cousin marriage), and environmental exposures (no, yes). Positive family history was considered when the PCOS occurred in other family members, i.e., sister, mother, aunt or any other close relative of the participant. For environmental risk factors, exposures to pesticides, herbicides, and industrial effluents were considered.

### Ethical Approval:

The Research Ethics Committee of Women University Multan gave the formal approval of the study (No: WUM/UREC/0008; Dated: October 28, 2020). All participants provided informed consent, in line with the principles outlined in the Helsinki-II declaration

### Genotyping and molecular methods:

Blood samples were obtained from cases and controls. Serum adiponectin levels were estimated by ELISA using Invitrogen Human Adiponectin Kits (Cat.# KHP0041, ThermoFisher Scientific). Genomic DNA was extracted by the salt precipitation method. SNPs rs1501299 and rs17300539 in *ADIPOQ* were genotyped by ARMS-PCR and RFLP methods, respectively (as per^12^). For rs1501299, PCR was done in a final reaction mixture volume of 20 μL, containing genomic DNA (100 ng), 1X Taq buffer, each primer (0.4 μM), MgCl_2_ (2 mM), 250 μM/ 0.25 μM dNTPs (Cat.# R0182), 1U of *Taq* DNA polymerase (Cat.# EP0402) and sterile water. For rs17300539, every reaction mixture final volume of 25 μL, consisted of Taq buffer (1X), MgCl_2_ (2 mM), dNTPs (250 μM/ 0.25 μM), each primer (1.0 μM), *Taq* polymerase (1U) and 100 ng DNA.

The amplicons of rs1501299 were run on 2% agarose gel. However, the PCR products of rs17300539 were analyzed by 1.5% agarose gel electrophoresis and digested with *Msp*I restriction endonuclease (ThermoFisher Scientific, Cat.# ERO541) overnight at 37°C. Finally, the digested products were separated on 2% agarose gel electrophoresis.

### Statistical analysis:

Data were analyzed using SPSS (v-20.0). Descriptive statistics was employed and logistic regression was applied in order to find the risk factors. For comparisons of more than two means, a one-way ANOVA and post-hoc multiple comparison were carried out. For both SNPs, the genotypic and allelic frequencies were calculated for PCOS and controls according to Hardy-Weinberg equilibrium (HWE). The relationship between SNPs and PCOS pathogenesis was analyzed by inheritance models like codominant, dominant, recessive, and over-dominant. Haplotypes were generated manually.

## RESULTS

### Sample characteristics, demographic and risk attributes:

The demographic attributes and risk factors for PCOS and the control population are presented in Table-I. The odds of PCOS were higher in women with a higher age category; however, the differences were statistically non-significant. With respect to ethnicity, Saraikis were more susceptible to the development of PCOS (OR= 6.23; *p*= 0.002) with reference to Urdu speaking individuals.

**Table-I:** Sample characteristics, demographic attributes and risk factors for PCOS and controls.

Parameter	PCOS	Controls	χ2	OR (95%CI)	p-value
	n (%)	n (%)	p-value		
***Age groups (years)***					
I (15-30)	137 (67)	101 (67)	0.686	1.00 (Ref.)
II (>30-45)	62 (30)	43 (29)		1.73 (0.50-6.03)	0.39
III (>45-60)	5 (3)	6 (4)		1.63 (0.48-5.48)	0.432
***Ethnicity***					
Urdu	4 (2)	14 (9)	<0.001***	1.00 (Ref.)
Punjabi	15 (7)	32 (21)		1.64 (0.46-5.84)	0.445
Saraiki	185 (91)	104 (70)		6.23 (1.99-19.41)	0.002**
***Obesity (BMI Kg/m^2^)***					
Underweight (<17.9)	3 (2)	9 (6)	<0.001***	1.00 (Ref.)
Normal (18-25.9)	52 (25)	111 (74)		1.41 (0.37-5.41)	0.621
Overweight (26-29.9)	63 (31)	17 (11)		11.12 (2.71-45.64)	0.001***
Obese (30 and above)	86 (42)	13 (9)		19.85 (4.75-83.01)	<0.001***
Serum adiponectin (μg/mL)	2.41±0.15	3.05±0.48		0.103
***History of smoking***					
Non-smoker	157 (77)	129 (86)	0.03*	1.00 (Ref.)	
Passive smoker	47 (23)	21 (14)		1.81 (1.03-3.19)	0.04*
***Family history***					
Negative	138 (68)	146 (97)	<0.001***	1.00 (Ref.)	
Positive	66 (32)	4 (3)		17.46 (6.20-49.18)	<0.001***
***Consanguinity***					
Unrelated marriages	19 (15)	19 (31)	0.008**	1.00 (Ref.)	
Cousin marriages	111 (85)	42 (69)		2.64 (1.28-5.48)	0.009**
***Exposure to pollutants***					
No	183 (90)	146 (97)	0.006**	1.00 (Ref.)	
Yes	21 (10)	4 (3)		4.19 (1.41-12.47)	0.010**

* p≤0.05=significant;

** p≤0.01=highly significant;

*** p≤0.001=very highly significant;

OR, odds ratio; CI, confidence interval

Obesity was highly prevalent among the PCOS subjects (*p*<0.001) and logistic regression analysis indicated that obese and overweight women were more prone to PCOS. Serum adiponectin level was low in PCOS women (2.41±0.15 vs. 3.05±0.48 μg/mL) as compared to controls, but the differences were statistically not significant (*p*=0.103).

The difference in smoking history was observed to be statistically significant (*p*=0.03) and passive smokers showed a greater risk of syndrome relative to non-smokers. Family history of PCOS indicated a highly significant difference in PCOS cases versus controls (*p*<0.001), i.e., 32.4% of cases had a positive family history of PCOS compared to 2.7% of controls who had a positive history. Regression analysis revealed that the patients with positive family history were more susceptible to disease (*p*<0.001). The individuals with parental consanguinity had 2.6 times higher risk of PCOS as compared to individuals without parental consanguinity (*p*=0.009). The women exposed to pollutants showed significantly higher odds (OR=4.19; *p*=0.010) of PCOS relative to unexposed women.

### Genetic analyses of SNP rs1501299:

At the rs1501299 locus, G allele was more frequent in PCOS cases (85% vs. 72%), while T allele was more frequent among controls (28% vs. 15%), and the differences were statistically significant (*p*<0.001; Table-II). With respect to inheritance models, the genotypes at rs1501299 were significantly associated with PCOS risk in the co-dominant model; individuals with GG genotype (OR=2.31; *p*=0.031) showed high risk relative to the genotype TT (*p*=0.031). Recessive and over-dominant models showed a significant association with PCOS (*p*<0.001). The distribution of genotypes in PCOS and controls deviated from HWE (*p*<0.001, *p*=0.034; respectively).

**Table-II:** Genotypic and allelic frequencies for SNP rs1501299 in *ADIPOQ.*

Genotypes	PCOS	Controls	χ2	OR (95%CI)	p-value
	n (%)	n (%)	ps-value		
***Co-dominant model***					
GG	152 (76)	80 (54)	<0.0001***	2.31 (1.08-4.92)	0.031*
GT	33 (17)	49 (34)		0.82 (0.36-1.89)	0.636
TT	14 (7)	17 (12)		1.00 (Ref.)	
Total	199	146			
***Allelic model***					
G	337 (85)	209 (72)	<0.0001***	2.19 (1.51-3.19)	<0.0001***
T	61 (15)	83 (28)		1.00 (Ref.)	
Total	398	292			
***Dominant model***					
TT	14 (7)	17 (12)	0.139	1.00 (Ref.)	
GT+GG	185 (93)	129 (88)		1.74 (0.83-3.66)	0.143
***Recessive model***					
GT+TT	47 (24)	66 (45)	<0.001***	1.00 (Ref.)	
GG	152 (76)	80 (55)		2.67 (1.68-4.23)	<0.001***
***Over-dominant model***					
GT	33 (17)	49 (34)	<0.001***	1.00 (Ref.)	
GG+TT	166 (83)	97 (66)		2.37 (0.24-0.65)	<0.001***

* =statistically significant;

*** =very highly significant

### Genetic analyses of SNP rs17300539:

At rs17300539 locus, allele A was observed to be more frequent in PCOS women compared to controls, who showed a high occurrence of G allele (*p*=0.139; Table-III). The genotypes were significantly associated with elevated risk of PCOS in the co-dominant model (*p*=0.049), and AG genotype had higher odds among the patients (OR=1.98; *p*=0.014) relative to the reference genotype GG. In the dominant model, genotype AG+AA (OR=1.89; *p*=0.018); and in over-dominant model (OR=0.57; *p*=0.026). The genotypic distribution in PCOS and controls was concordant with HWE. Haplotype analyses revealed that G-G was most frequent on the forward strand (80%), whereas G-A was observed to be most prevalent on the reverse strand (52%; *p*=0.011) (Table-IV).

**Table-III:** Genotypic and Allelic frequencies for SNP rs17300539 in *ADIPOQ.*

Genotypes	PCOS	Controls	χ2	OR (95%CI)	p-value
	n (%)	n(%)	p-value		
***Co-dominant model***					
GG	43 (23)	36 (36)	0.049*	1.00 (Ref.)	
AG	125 (66)	53 (53)		1.98 (1.14-3.41)	0.014*
AA	20 (11)	11 (11)		1.52 (0.64-3.60)	0.338
Total	188	100			
***Allelic model***					
G	211 (56)	125 (62)	0.139	1.00 (Ref.)	
A	165 (44)	75 (38)		1.30 (0.92-1.85)	0.139
Total	376	200			
***Dominant model***					
GG	43 (23)	36 (36)	0.017*	1.00 (Ref.)	
AG+AA	145 (77)	64 (64)		1.89 (1.11-3.29)	0.018*
***Recessive model***					
AG+GG	168 (89)	89 (89)	0.925	1.00 (Ref.)	
AA	20 (11)	11 (11)		0.96 (0.44-2.10)	0.925
***Over-dominant model***					
AG	125 (67)	53 (53)	0.025*	1.00 (Ref.)	
GG+AA	63 (33)	47 (47)		0.57 (0.35-0.93)	0.026*

* =statistically significant

**Table-IV:** Haplotype analysis using rs1501299 and rs17300539.

Forward strand	PCOS, n	Controls, n	Total, n	Frequency (%)	χ2	p-value
G-G	152	75	227	80	0.725	0.696
G-A	20	10	30	11		
T-G	14	10	24	9		
T-A	-	1	1	0
***Reverse strand***						
G-A	108	38	146	52	11.2	0.011**
T-A	35	22	57	20		
G-G	31	21	52	18		
T-G	12	15	27	10		

** =statistically significant

The association of serum adiponectin levels with genotypes of rs1501299 in PCOS and healthy controls was checked and it showed statistically significant differences (Table-V). For rs17300539, the differences in the serum adiponectin levels in PCOS and controls were statistically non-significant (*p*= 0.751).

**Table-V:** Association of serum adiponectin level with genotypes of rs1501299 in PCOS and controls.

Serum Adiponectin	(μg/mL)	SNP	Genotype	PCOS	Controls	F-value	p-value
				(Mean± SEM)	(Mean± SEM)		
		rs1501299	GG	2.17±0.14	1.81±0.34	5.75	<0.001***
			GT	2.76±0.52	4.54±0.97		
			TT	4.08±0.77	4.40±2.40		

## DISCUSSION

There are scarce data regarding the role of adiponectin gene variants in pathogenesis of PCOS, particularly for the Pakistani population. This is a pilot study elucidating the potential association of SNPs in *ADIPOQ* in the predisposition to PCOS in the population of Southern Punjab, Pakistan. In the current study, certain factors like obesity, smoking, family history, consanguinity, exposure to pollutants, and ethnicity were significant risk predictors in the pathogenesis of PCOS.

In this study, obesity was strongly associated with PCOS (p<0.001), consistent with extensive literature linking adiposity to insulin resistance and hyperandrogenism in PCOS.^6^,^13^,^14^. The observed lower adiponectin levels in PCOS women (though statistically non-significant, p=0.103) align with studies showing hypoadiponectinemia as a potential contributor to metabolic dysfunction in PCOS.^6^,^7^ The lack of significance may be due to small sample size or confounding metabolic factors. Smoking was more prevalent in patients compared to controls (*p*=0.03). Glintborg et al. also reported a higher smoking rate in PCOS women.^15^

While age is a known factor in PCOS manifestation, this study found a non-significant trend toward higher PCOS odds in older women. This contrasts with some of the previous studies suggesting that PCOS symptoms often emerge in adolescence and young adulthood but may persist with age.^16^ The non-significant association here may reflect population-specific variations or differences in diagnostic criteria.

In the current study, there was significantly higher PCOS risk among Saraiki women (OR=6.23, p=0.002) compared to Urdu-speaking individuals, which underscores the role of ethnicity in PCOS susceptibility. Previous studies have demonstrated ethnic disparities in PCOS prevalence and severity, possibly due to genetic, dietary, or lifestyle differences.^3^,^5^ This finding highlights the need for population-specific risk assessments.

Positive family history was a statistically significant risk predictor of PCOS, which is concordant with Al-Khaduri et al.^17^ who witnessed an association of family history with PCOS. The current study population indicated that consanguineous marriages exhibited a significant risk of PCOS relative to outer family marriages. Here, parental consanguinity further elevated PCOS risk (OR=2.6, p=0.008), suggesting recessive genetic contributions, which aligns with research implicating rare homozygous variants in consanguineous populations.^5^,^17^ The current study reported that women exposed to environmental pollutants like herbicides, pesticides, and industrial effluents, exhibited higher risk of PCOS compared to control group. These findings are concordant with Lin et al.^18^

The current study revealed a statistically significant association between rs1501299 and rs17300539 in Pakistani PCOS women. Similar results were reported for Korean and Chinese populations where rs1501299 was associated with risk of developing PCOS.^19^ Xian et al.^8^ revealed a significant association with PCOS. However, a meta-analysis of the Asian population conducted by Tiongco et al.^20^ indicated that individuals with rs1501299 polymorphisms were less susceptible to PCOS. Sun et al.^21^ showed a significant association between rs17300539 and PCOS in the Chinese population, which is concordant to our study. Few studies revealed that SNPs located in promoter region (rs17300539) exhibited the strongest association with serum adiponectin levels.^22^ The current study findings revealed a highly significant pattern of serum levels of adiponectin in genotypes of rs1501299 in PCOS.

### Limitations

First, only two SNPs have been evaluated in this research. Due to sources constraints, we limited this study to two SNPs only. For SNPs rs1501299, the distribution of genotypes in PCOS and controls deviated from HWE may suggest possible population stratification, or selection bias. In these analyses, obesity, family history, and pollutant exposure were significant but may not have been fully adjusted for in genetic analyses, potentially skewing SNP associations. Finally, a large sample size with several SNPs would be ideal to draw a more comprehensive picture of risk estimation of PCOS in this population. The replication of this study design in independent cohorts with deeper genomic coverage would be more prudent.

Despite its limitations, the study has several notable strengths that enhance its scientific contribution to PCOS research, particularly in underrepresented population. To the best of our knowledge, no such study has been done in Southern Punjab. This study highlights ethnic-specific risks (e.g., Saraiki women had 6.23 times higher PCOS odds), emphasizing the need for diverse genetic research. Further, this study examines parental consanguinity revealing 2.6 times higher PCOS risk, which is underreported in Western studies.

## CONCLUSION

This study demonstrates that rs1501299 and rs17300539 in *ADIPOQ* gene are associated with an increased vulnerability of Southern Punjab Pakistani women to PCOS. Additionally, haplotype analysis showed that G-A haplotype was more prevalent in the reverse strand, whereas in the forward strand, the G-G haplotype was more common. Thus, the current study may help set the stage for further research on gynecological malignancy, infertility, and its long-term effects. To further understand the relevance of these risk variables, more research in this area with a larger sample size from ethnically varied groups may be beneficial.

### Authors’ Contribution:

**AM:** Designed the study, collected data, interpreted and drafted manuscript.

**SM:** Statistical analysis, critically analyzed the manuscript. Critical Review.

**AB** and **NK:** Supervised the study. Responsible and accountable for the accuracy and integrity of data.

All authors have read and approved the final version of the manuscript.
